# The Dose Makes the Poison: Perturbative Steps toward the Ultimate Linearized Coupled Cluster Method

**DOI:** 10.1021/acs.jctc.6c00366

**Published:** 2026-04-22

**Authors:** Sylvia J. Bintrim, Ella R. Ransford, Kevin Carter-Fenk

**Affiliations:** Department of Chemistry, 6614 University of Pittsburgh, Pittsburgh, Pennsylvania 15218, United States

## Abstract

“Addition-by-subtraction” coupled cluster (CC) approaches provide a promising approach to treating the difficult strong correlation problem by simplifying the standard CC equations. In a separate vein, linearized CC methods have drawn interest for their lower computational cost, increased parallelizability, and favorable properties for extension to the excited state–but the inclusion of ring/crossed-ring terms causes singularities even for single bond breaking. A linearized, addition-by-subtraction CC method called linearized ladder CCD (linLCCD) removes these terms to avoid divergences, but linLCCD underestimates dynamical correlation. Herein we resolve this deficiency of linLCCD by introducing a linearized external coupled cluster perturbation theory that adds a second-order ring/crossed-ring correction back into a linLCCD reference wave function. Our resultant xlinCCD(2) method is regular and yields comparable results to linearized CCD in weakly correlated regimes.

## Graphical abstract

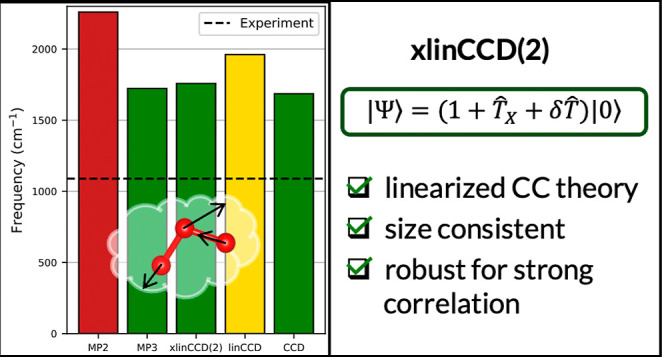

## Introduction

Strongly correlated systems (e.g., transition metal complexes or molecules undergoing bond dissociation) are exceedingly difficult to accurately and affordably simulate using quantum chemistry methods. In such systems, no single electron configuration dominates the wave function; instead, the wave function is comprised of multiple (or many) nearly degenerate configurations of roughly equal weights. Full configuration interaction (FCI) offers the most straightforward solution to the strong correlation problem by expanding the wave function in the complete basis of all Slater determinants, a procedure which is exact but scales exponentially with system size. Due to its high cost, practitioners are forced into the nontrivial selection of an “active space” of important orbitals for FCI-based methods.^1–4^ While there are notable efforts to automate the selection of such orbitals,^5–8^ the choice of active space remains a source of uncontrolled error.^9^ In an effort to simultaneously avoid active space selection and reduce computational cost, our group has been pursuing novel, single-reference coupled cluster (CC) methods that capture the qualitative essence of strong correlation at polynomial cost. Furthermore, we have shown that improvements to the CC ground state translate to improvements in the excited states of strongly correlated systems.^10^

One seemingly paradoxical line of modern inquiry into treating the strong correlation problem in single-reference CC is the simplification of the CC equations by removal of problematic components of the wave function.^11^ Such simplifications lead to a family of approximations known as addition-by-subtraction CC. Examples of such approaches include the distinguishable cluster approximation, in which exchange couplings between doubles clusters are neglected, allowing for smooth dissociation of dinitrogen.^12–14^ There has also been a recent surge of interest in seniority-zero CC approaches such as the pair coupled cluster doubles (pCCD)^11,15–20^ approximation, in which only paired double substitutions contribute to the CC wave function, yielding highly affordable, single-reference methods that are robust in cases of static correlation.^21–25^ Despite the formal $O\left(N^{3}\right)$ scaling of pCCD (or equivalently, antisymmetric product of 1-reference orbital geminals), it is not invariant to unitary transformations within the occupied or virtual orbitals, requiring orbital optimization and localization to achieve size-consistent results.^17,26–29^

Singlet-paired and triplet-paired CCD (CCD0 or CCD1, respectively) methods decouple the singlet- and triplet-paired doubles amplitudes in efforts to attain similar reliability to pCCD while maintaining orbital invariance.^30^ Perturbative recouplings (CCD with frozen singlet- or triplet-paired amplitudes [CCDf0/CCDf1]) can be introduced by fully optimizing one set of amplitudes in the presence of the frozen amplitudes of the other.^10,31^ However, it remains unclear why the decoupling of singlet- and triplet-paired amplitudes in CCD0/CCD1 (and hence CCDf0/CCDf1) helps to avoid the failures of CCD and CCSD in strongly correlated systems.

In contrast, simplifications to the CC equations that use diagrammatic arguments to precisely target terms for removal have a clear physical significance.^30,32^ One example is ring-CCD (or the particle-hole random phase approximation), which removes terms associated with ladder diagrams and typically also exchange interactions to achieve somewhat better dissociation limits for chemical bonds.^33–36^ Ladder-CCD or, equivalently, the particle–particle random phase approximation, is known to perform well for the low-density uniform electron gas (where electron–electron interactions are poorly screened and thus strong), providing some physical explanation for its success in strongly correlated systems.^35,37–45^

Recently, one of us applied the philosophy of addition-by-subtraction CC in conjunction with diagrammatic arguments to improve the robustness of linearized CCD (linCCD) in strongly correlated cases. While linCCD itself does not fall under the addition-by-subtraction umbrella, it offers several advantages, including a straightforward variational framework, simpler derivatives, and improved parallel efficiency.^46–48^ Despite these advantages, linCCD displays catastrophic divergences in strongly correlated systems.^49^ While prior work involved regularizing the linCCD equations to suppress small energy-gap denominators,^47^ our recent investigations instead suggest that ring and crossed-ring terms are to blame.^50^ Removing the offending diagrams results in an addition-by-subtraction theory called linearized ladder CCD (linLCCD)^50^ which is robust for strongly correlated systems and has the favorable properties of unitary invariance and size consistency. Despite the qualitative robustness of linLCCD in strongly correlated systems, it lacks quantitative accuracy, missing particle-hole screening typically supplied by ring and crossed-ring terms.

In this work, we improve upon linLCCD with a linearized external coupled cluster perturbation theory (xCCPT)^51^ correction to reintroduce this missing correlation energy. The resultant approach, which we call second-order external linearized CCD [xlinCCD(2)], incorporates all forms of linCCD correlation (driver, ladder, ring, and crossed-ring terms), and performs well for strongly correlated systems without sacrificing dynamical correlation. Importantly, we choose a partitioning of the Hamiltonian that dresses the electron-repulsion integrals and one-particle energies with correlation from the reference linLCCD wave function, stabilizing the addition of the ring and crossed-ring terms that typically cause the divergence of infinite-order linCCD. In fact, our results feature cases, such as the dissociation curve of dinitrogen, where the perturbative ring/crossed-ring terms and dressed one-particle energies actually prevent divergence of the parent linLCCD theory. As our xlinCCD(2) method contains all types of correlation present in linCCD but often produces results of comparable accuracy to CCD in cases where linCCD diverges, we believe xlinCCD(2) is perhaps the most complete linearized coupled cluster doubles theory to date. Given the widespread use of linCCD^19,25,28,52–55^ or configuration interaction doubles^56^ in combination with pCCD, as well as multireference linCCD,^57–59^ we expect that our results may encourage similar applications of xlinCCD(2).

## Theoretical Background

Throughout this work, occupied orbitals will be indexed as {*i*, *j*, *k*, *l*, ... } and virtual orbitals as {*a*, *b*, *c*, *d*, ... }.

The standard CCD approach employs an exponential ansatz for the wave function

1 $$|\Psi_{CC}\rangle =e^{{\mathrm{T̂}}_{2}}|\Phi_{0}\rangle$$

where |Φ_0_⟩ is usually the Hartree–Fock ground state reference determinant

2 $${\mathrm{T̂}}_{2}=\frac{1}{4}\sum_{ijab}t_{ij}^{ab}{â}_{a}^{†}{â}_{b}^{†}{â}_{j}{â}_{i}$$

is the double-substitution operator, and ${â}_{i}$ and ${â}_{a}^{†}$ are particle annihilation and particle creation operators, respectively. The energy and amplitude equations for CCD are

3a $$E=\langle \Phi_{0}|e^{-{\mathrm{T̂}}_{2}}Ĥe^{{\mathrm{T̂}}_{2}}|\Phi_{0}\rangle =\frac{1}{4}\sum_{ijab}t_{ij}^{ab}\langle ij||ab\rangle$$

3b $$\langle \Phi_{ij}^{ab}|e^{-{\mathrm{T̂}}_{2}}Ĥe^{{\mathrm{T̂}}_{2}}|\Phi_{0}\rangle =0$$

where |Φ_*ij*_^*ab*^⟩ is a doubly excited determinant.

In linearized CCD (linCCD), we truncate the ansatz at first order in the Taylor expansion of $e^{{\mathrm{T̂}}_{2}}$ , giving

4 $$|\Psi_{CC}\rangle =\left(1+{\mathrm{T̂}}_{2}\right)|\Phi_{0}\rangle$$

Whereas variational CC methods generally lead to nonterminating series,^47,60^ we note that the linCCD energy functional can be written in Hermitian form

5 $$E=\langle 0|{\left[\left(1+{\mathrm{T̂}}_{2}^{†}\right)Ĥ\left(1+{\mathrm{T̂}}_{2}\right)\right]}_{SC}|0\rangle$$

where “SC” denotes strongly connected diagrams.^46,47^ Varying this expression with respect to ${\mathrm{T̂}}_{2}^{†}$ leads to the following doubles amplitude equation for linCCD at stationarity in the spin–orbital basis

6 $$\begin{array}{ll} 0= & v_{ij}^{ab}-P_{ij}\left(t_{kj}^{ab}f_{i}^{k}\right)+P_{ab}\left(f_{c}^{a}t_{ij}^{cb}\right)\\ & +\frac{1}{2}t_{kl}^{ab}v_{ij}^{kl}+\frac{1}{2}v_{cd}^{ab}t_{ij}^{cd}\\ & +P_{ij}P_{ab}\left(v_{ic}^{ak}t_{kj}^{cb}\right) \end{array}$$

where we have employed the Einstein summation convention, *v*_pq_^rs^ are antisymmetrized two-electron integrals ⟨*rs*||*pq*⟩ and $P_{pq}=1-\left(p↔q\right)$ are index permutation operators. The connection of each term to a class of Feynman diagrams is as follows: The first three terms are known as “driver” terms, terms four and five correspond to “ladder” diagrams, and the final term encompasses “ring” and “crossed-ring” diagrams. Thus, omitting the final term leads to the linLCCD equations, omitting the final term and the particle–particle ladder term (term 5) gives the so-called hole–hole approximation to linLCCD [linLCCD­(hh)], and eliminating everything but the driver terms yields the second-order Møller–Plesset perturbation theory (MP2) equation. For a detailed analysis of each term in the linCCD equations along with the corresponding diagrams, we refer the interested reader to reference 50, and for an overview of each diagram in the CCD equations we suggest reference 61.

xCCPT perturbatively includes missing components of the (full) cluster operator T̂ on top of an initial CC calculation that uses a (potentially incomplete) “external” ${\mathrm{T̂}}_{X}$ cluster operator.^51^ Here, we introduce the linearization of the xCCPT equations for the first time. Let ${\mathrm{T̂}}_{X}$ correspond to a linearized CCD starting point such as linLCCD. We can partition the Hamiltonian into a one-electron part ${Ĥ}_{0}$ and fluctuation potential V̂

7 $$Ĥ={Ĥ}_{0}+\lambda \mathrm{V̂}$$

and define

8 $$\mathrm{T̂}={\mathrm{T̂}}_{X}+\delta \mathrm{T̂}={\mathrm{T̂}}_{X}+\sum_{k}\lambda^{k}\delta {\mathrm{T̂}}^{\left(k\right)}$$

where *k* denotes the order of the xCCPT correction to the wave function. We choose^62^

$$\begin{array}{ll} {Ĥ}_{0} & =\underset{ik}{\sum }\left(f_{i}^{k}a_{i}^{†}a_{k}+\frac{1}{2}\underset{jab}{\sum }t_{Xij}^{\;\;ab}\left\langle kj\right||ab\rangle a_{i}^{†}a_{k}\right)\\ & +\underset{ac}{\sum }\left(f_{c}^{a}a_{a}^{†}a_{c}-\frac{1}{2}\underset{ijb}{\sum }t_{Xij}^{\;\;cb}\left\langle ij\right||ab\rangle a_{a}^{†}a_{c}\right) \end{array}$$ 9

and modify $\mathrm{V̂}=Ĥ-{Ĥ}_{0}$ accordingly. This choice of Hamiltonian partitioning dresses the one-particle energies with correlation from the reference wave function (e.g., linLCCD), which has an important effect on the stability of the resultant perturbation theory (see Figure S1).

We will also let $\mathrm{X̂}={\mathrm{X̂}}_{0}+{\mathrm{X̂}}_{V}$ where

10 $${\mathrm{X̂}}_{0}=\left(1-{\mathrm{T̂}}_{X}\right){Ĥ}_{0}\left(1+{\mathrm{T̂}}_{X}\right)\approx {Ĥ}_{0}+\left[{Ĥ}_{0},{\mathrm{T̂}}_{X}\right]$$

and

11 $${\mathrm{X̂}}_{V}=\left(1-{\mathrm{T̂}}_{X}\right)\lambda \mathrm{V̂}\left(1+{\mathrm{T̂}}_{X}\right)\approx \lambda \mathrm{V̂}+\left[\lambda \mathrm{V̂},{\mathrm{T̂}}_{X}\right]$$

are linearized, similarity-transformed ${Ĥ}_{0}$ and V̂ operators. Having chosen our Hamiltonian partitioning and reference wave function, we now derive the first order perturbative correction to the wave function and second order correction to the energy via xCCPT.

We begin by writing the linCCD equation for the first order correction to the doubles amplitudes $\delta \mathrm{T̂}≔\lambda \delta {\mathrm{T̂}}^{\left(1\right)}$

12 $$\langle \Phi_{ij}^{ab}|\left(1-\lambda \delta \mathrm{T̂}\right)\mathrm{X̂}\left(1+\lambda \delta \mathrm{T̂}\right)|0\rangle \approx \langle \Phi_{ij}^{ab}|\left(\mathrm{X̂}+\left[\mathrm{X̂},\lambda \delta \mathrm{T̂}\right]\right)|0\rangle =0$$

This simplifies to

13 $$\langle \Phi_{ij}^{ab}|\left(\left[{Ĥ}_{0},{\mathrm{T̂}}_{X}\right]+\lambda \mathrm{V̂}+\left[\lambda \mathrm{V̂},{\mathrm{T̂}}_{X}\right]\right)|0\rangle +\langle \Phi_{ij}^{ab}|\left[{Ĥ}_{0},\lambda \delta \mathrm{T̂}\right]|0\rangle =0$$

when retaining only linear nonzero terms and truncating the perturbation series at λ^1^. Recalling that the expression for $\delta {\mathrm{T̂}}^{\left(1\right)}$ is analogous to eq 2, when λ = 1 eq 13 implies

14 $$0=X_{ij}^{ab}+P_{ab}\left(X_{e}^{b}\delta t_{ij}^{ae}\right)-P_{ij}\left(X_{j}^{m}\delta t_{im}^{ab}\right)$$

where

$$\begin{array}{ll} X_{ij}^{ab} & =v_{ij}^{ab}+\frac{1}{2}t_{Xmn}^{\;\;\;ab}v_{ij}^{mn}+\frac{1}{2}t_{Xij}^{\;\;ef}v_{ef}^{ab}+P_{ij}P_{ab}\left(t_{Xim}^{\;\;ae}v_{ej}^{mb}\right)\\ & +P_{ab}\left(f_{e}^{b}t_{Xij}^{\;\;ae}\right)-P_{ij}\left(f_{j}^{m}t_{Xim}^{\;\;ab}\right) \end{array}$$ 15

can be interpreted as a set of electron repulsion integrals that are screened by correlation effects from the reference wave function, and

$$X_{b}^{a}=f_{b}^{a}-\frac{1}{2}t_{Xmn}^{\;\;\;\;ae}v_{be}^{mn}$$ 16a

$$X_{i}^{j}=f_{i}^{j}+\frac{1}{2}t_{Xim}^{\;\;\;\;ef}v_{ef}^{jm}$$ 16b

are dressed one-particle energies resulting from our choice of ${Ĥ}_{0}$ that bear a resemblance to correlated orbital energies used in other approaches.^32,62,63^ If ${\mathrm{T̂}}_{X}$ comes from a converged linLCCD calculation, then

$$0=v_{ij}^{ab}+\frac{1}{2}t_{Xmn}^{\;\;\;\;ab}v_{ij}^{mn}+\frac{1}{2}t_{Xij}^{\;\;\;\;ef}v_{ef}^{ab}+P_{ab}\left(f_{e}^{b}t_{Xij}^{\;\;ae}\right)-P_{ij}\left(f_{j}^{m}t_{Xim}^{\;\;ab}\right)$$ 17

holds, and the first order amplitude correction eq (eq 14) simplifies to

18 $$0=P_{ij}P_{ab}\left(t_{Xim}^{ae}v_{ej}^{mb}\right)+P_{ab}\left(X_{e}^{b}\delta t_{ij}^{ae}\right)-P_{ij}\left(X_{j}^{m}\delta t_{im}^{ab}\right)$$

where term 1 reintroduces the heretofore missing ring/crossed-ring correlation and the last two, mosaic-style/disconnected terms^32,64^ couple ring/crossed-ring correlation to ladder and driver components. Note that in the limit that *T*_X_ comes from linCCD, *X*_*ij*_^*ab*^ = 0 and so δT̂ = 0, as expected.

Finally, for xlinCCD(2), the second order energy correction is simply

19 $$\delta E^{\left(2\right)}=\frac{1}{4}\sum_{ijab}\langle ij||ab\rangle \delta t_{ij}^{ab}$$

and to first order, the wave function is

20 $$|\Psi \rangle \approx |\Psi^{\left(0\right)}\rangle +|\Psi^{\left(1\right)}\rangle =\left(1+{\mathrm{T̂}}_{X}\right)|0\rangle +\delta \mathrm{T̂}|0\rangle =\left(1+{\mathrm{T̂}}_{X}+\delta \mathrm{T̂}\right)|0\rangle$$

## Computational Details

All calculations reported here use locally modified versions of Q-Chem v6.2 ^65^ or the PySCF software package.^66,67^ Dissociation curves were computed in the aug-cc-pVTZ basis^68–70^ and make use of both packages, while Hubbard model calculations were performed in PySCF. W4-11 calculations were carried out in Q-Chem.

Geometries and benchmark thermochemical energies are taken from the non-multireference subset of the W4-11 thermochemical database.^71^ To reduce errors from spin-contamination in the energies, we used self-consistently converged restricted open-shell Hartree–Fock (ROHF) orbitals to build the unrestricted Fock matrix for input into the unrestricted CC equations.^72^ To extrapolate our results to the complete basis set (CBS) limit, we use a two-point *n* ^–3^ extrapolation of the correlation energies with *n* = 3, 4 for the aug-cc-pVnZ basis sets.^73^

Ozone vibrational frequencies were calculated using MP and CC methods in Q-Chem in the aug-cc-pVDZ basis set. The geometries and vibrational frequencies for linCCD, CCD, MP3, and xlinCCD(2) approaches were computed via finite difference whereas CCSD analytic gradients were used. The SCF and CC amplitude convergence tolerances were set to 10^–10^.

Singlet–triplet (S–T) gaps of transition-metal diatomics were calculated via ΔMP and ΔCC methods in Q-Chem in the def2-QZVPPD^74,75^ basis (no frozen core approximation). Herein, we report TinySpins25: A set of theoretical best-estimates for S–T gaps of 25 heteronuclear diatomic first- and second–row transition metal complexes. Details on the composition of TinySpins25 can be found in the Supporting Information. In brief, the S–T gaps in TinySpins25 were calculated using the MRCC^76^ software package at the CCSDT­(Q)_Λ_ level with two-point extrapolation to the complete basis set limit. As validation of this choice, which was motivated by the findings of,^77^ we compared 7 complexes computed at the def2-TZVPPD level to the triple-ζ FCI best estimates in the Quest #8 data set and find a mean absolute error of just 0.05 eV.

Li^+^/ethylene carbonate (EC) cluster association energies Δ*U*_assoc_ = *U*_Li_*x*_EC_*y*__-*xU*_Li^+^_-*yU*_EC_ were calculated in the def2-TZVPD basis with the resolution of the identity, using Q-Chem. For CC calculations with #EC≥3, a two- or three-point extrapolation with FNOs was used with natural orbital occupation thresholds of 99.50%, 99.75%, and 99.80%.^78,79^ By comparison to canonical CC results, the 99.50%/99.75% occupation threshold extrapolation errors were 2 and 5 kcal/mol for LiEC and LiEC_3_, respectively. Given that the benchmark DLPNO–CCSD­(T) data also may contain substantial localization error, we believe that these extrapolation errors are sufficiently small.^80^ Cluster geometries optimized using ωB97X-D3BJ/def2-TZVPD and DLPNO–CCSD­(T)/aug-cc-pVTZ association energy data were taken from reference 81

For the BDEs of first–row transition metal diatomics, we used ROHF with the exact-two-component (X2C) scalar relativistic approximation^82–85^ for input into unrestricted CC, extrapolating the SCF^86^ and correlation energies^87,88^ using def2-TZVPP and def2-QZVPP^89^ basis sets. Spatial symmetry was not utilized at the SCF or CC steps. Reference bond lengths, spin states, and BDEs were taken from references 90,91 . Spin-orbit coupling corrections from reference 91 were applied.

## Results & Discussion

Covalent bond breaking is a classic case where absolute near-degeneracy static correlation is encountered,^92^ resulting in neccessarily multireference character of the wave function. Here we analyze the performance of our single-reference xlinCCD(2) method in producing qualitatively correct dissociation curves for several small molecules.

We begin with H_2_ in Figure 1a, where linCCD diverges after 2 Å separation. While linLCCD produces a smooth dissociation curve, it is significantly under-correlated by comparison to CCD, even at the equilibrium geometry. We show dissociation curves from two varieties of xlinCCD(2): one built atop a linLCCD reference that we call xlinCCD(2)@linLCCD, and another that uses a linLCCD­(hh) reference, called xlinCCD(2)@linLCCD­(hh). The latter is potentially more affordable with a simple change of basis (see Supporting Information for details on its computational cost.). Compared with linLCCD, both xlinCCD(2) methods provide an equilibrium energy and a finite asymptotic dissociation energy closer to those of CCD, albeit with a dissociation barrier. The asymptotic limit of xlinCCD(2)@linLCCD is a near-perfect match to CCD, differing by only 0.5 kcal/mol. This small imperfection is absent in the minimal-basis dissociation curve shown in Figure S2, as xlinCCD(2)@linLCCD is exact in this limit. The results for the heterodiatomic FH dissociation curve in Figure 1b are qualitatively similar to H_2_.

**Figure 1:**
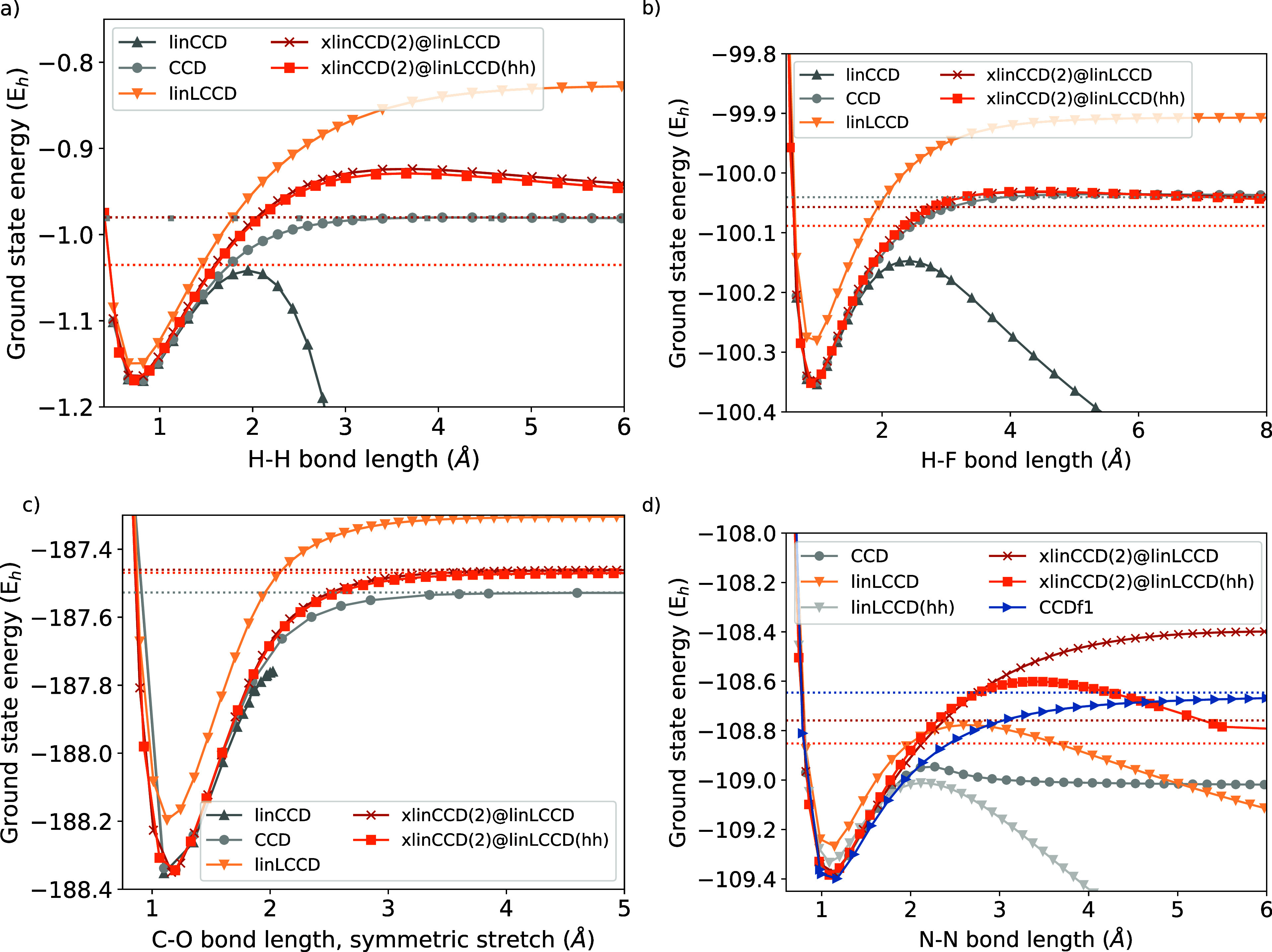
Ground state dissociation curves of (a) H_2_, (b) FH, (c) CO_2_ undergoing symmetric stretch, and (d) spatially symmetry-adapted N_2_ in the aug-cc-pVTZ basis with restricted orbitals. The dotted horizontal lines indicate asymptotic limits estimated at 100 Å, except for the case of CCDf1, where the limit was estimated at 12 Å.

Notably, the disconnected terms—which manifest directly as a result of our zeroth-order Hamiltonian partitioning—have a profound impact on the stability of xlinCCD(2), as shown in Figure S1 for the dissociation of FH molecule. Without these terms, the ladder and ring/crossed-ring coupling is entirely neglected, and the divergence of the parent linCCD theory is only marginally suppressed. Mosaic contributions prevent divergence by imbuing the one-particle energies with linearized particle–particle and/or hole–hole screening (depending on the reference wave function), which manifests as a gap-opening effect. As we will later see, the mosaic terms take the form of disconnected double substitutions and therefore improve the overall quality of multiple-bond dissociation curves.

For the symmetric dissociation of CO_2_, the results in Figure 1c suggest that the xlinCCD(2) methods find a slightly higher dissociation limit than CCD, which may be attributable to the lack of quadratic terms (disconnected quadruples) that are important in the dissociation of double bonds. While both are an upper bound to CCD, xlinCCD(2)@linLCCD­(hh) gives an energy at dissociation that is slightly closer to the CCD reference.

For the dissociation of the N_2_ triple bond (Figure 1d), no theory that truncates at doubles can be expected to provide quantitative accuracy.^93,94^ Our previous addition-by-subtraction CC method of choice,^10^ CCDf1, lacks the unphysical barrier produced by xlinCCD(2) or CCD. However, compared with CCDf1, xlinCCD(2) provides dissociation energies that are closer to the CCD result. Interestingly, xlinCCD(2) does not diverge, even though the underlying linLCCD and linLCCD­(hh) both dive downward for this triple bond. Of the two xlinCCD(2) methods, xlinCCD(2)@linLCCD­(hh) appears to give a better asymptotic energy, even though linLCCD­(hh) plunges downward more severely than linLCCD. Notably, very few (if any) methods that are linear in the wave function can dissociate spatial symmetry-adapted N_2_ without resorting to some form of regularization.^56^

Even for a transition metal diatomic like Cu_2_ (Figure 2), xlinCCD(2) avoids divergence in the dissociation limit and tracks the CCD curve reasonably well. Whereas linCCD diverges, xlinCCD(2) not only converges to a clear asymptotic limit but outperforms CCD in estimating the bond dissociation energy (BDE) relative to experiment.

**Figure 2:**
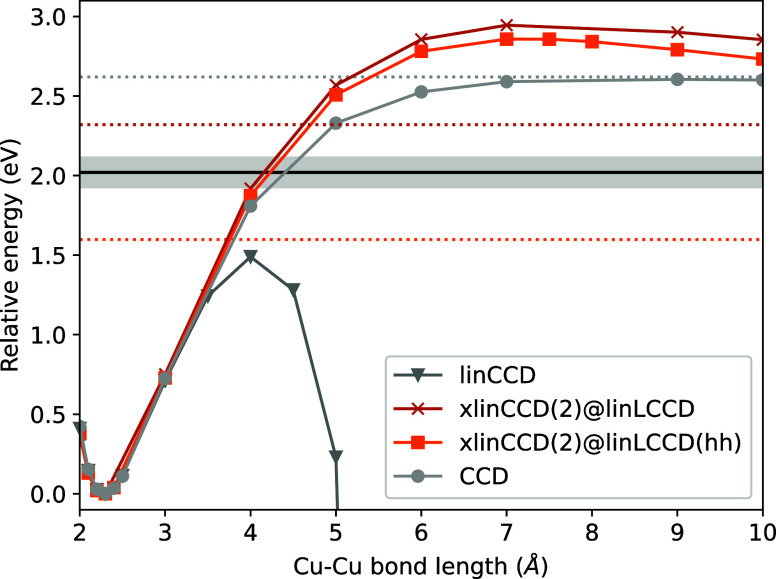
Ground state dissociation curves for Cu_2_ in the aug-cc-pVTZ basis with restricted orbitals. The dotted horizontal lines indicate the methods’ asymptotic limits calculated at 100 Å. The experimental dissociation energy is shown as a black line, with experimental uncertainty as a gray region.^95^

Our data for the Cu_2_ BDE inspired us to more thoroughly assess the performance of xlinCCD(2) for BDEs and other thermochemical data in the non-multireference subset of the W4-11 database.^71^ While these explicitly non-multireference thermochemical results do not directly probe the performance of xlinCCD(2) for strong correlation, our findings (Figure S3) reinforce that xlinCCD(2) consistently provides results of a quality comparable to those of linCCD or CCD at equilibrium geometries and less strongly correlated systems.

To assess the quality of potential energy surface shape provided by our methods within the Franck–Condon region, we computed frequencies of the three vibrational modes of ozone. The ground state ozone vibrational asymmetric stretch (highest-frequency mode) is known to be especially computationally challenging due to static correlation.^96,97^ For the ozone asymmetric stretch, Table 1 shows that the xlinCCD(2) methods perform noticeably better than linCCD and are on par with estimates from MP3 and CCD. The *t*_1_ amplitudes seem to play an important role in this vibrational mode, as we see much improvement in going from CCD to CCSD. This motivates further extension of xlinCCD(2) to include singles amplitudes in future work.

**Table 1:**
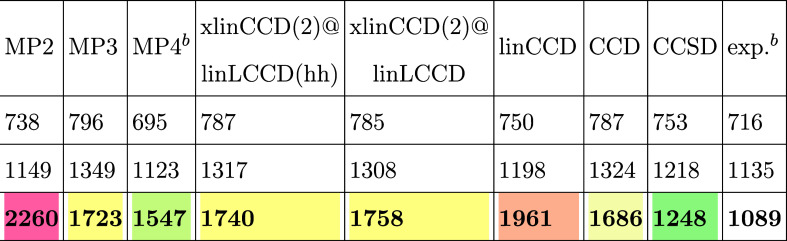
Harmonic Frequencies (cm^–1^) for the Vibrational Modes of Ozone^a^

a Our calculations were performed in the aug-cc-pVDZ basis.

b MP4 and experimental frequencies from.^96^

Next, we consider the Hubbard model,^98^ allowing us to directly modulate the interaction strength between electrons at different sites to assess the behavior of our methods in weak to strong correlation regimes. In Figure 3, we plot the ground state energy of a ten-site, one-dimensional, molecular Hubbard model at half-filling as a function of interaction strength (U/|*t*|). Our goal here is to assess how well various single-reference CC methods can capture the physics of the strongly correlated Hubbard model by tracking which methods can qualitatively reproduce the exact FCI result out to higher electron interaction strengths.

**Figure 3:**
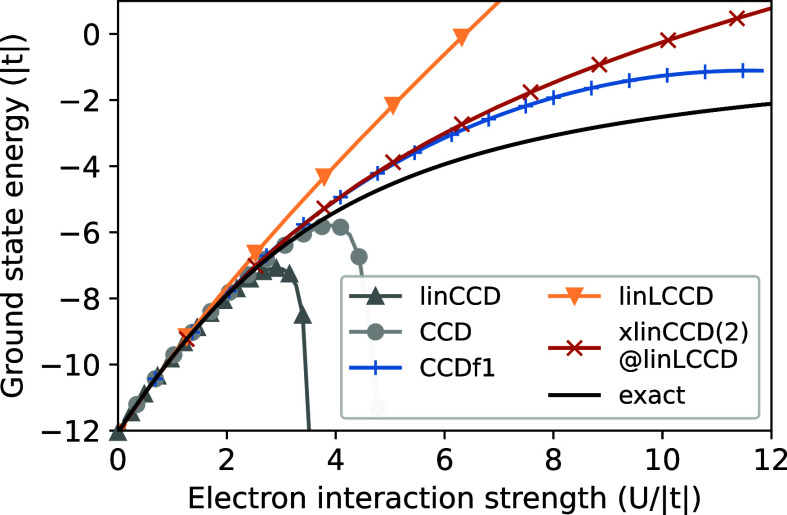
Ground state energies as a function of interaction strength (*U*/|*t* = – 1.5|) for a 10-site, half-filled Hubbard model with open boundary conditions. The exact reference is the FCI result.

Figure 3 shows that the linCCD energy diverges around *U*/|*t*| = 3 while CCD diverges slightly later, also monotonically decreasing with interaction strength, after *U*/|*t*|∼4. LinLCCD does not diverge downward but appears almost as under-correlated as Hartree–Fock.^10^ Although CCDf1 tracks the exact result more closely than xlinCCD(2)@linLCCD, the latter method does not “turn over” at high interaction strengths, making it potentially better-suited to serve as a ground state reference for excited state methods. Based on findings in our previous work, we suspect that if both ground and excited state methods avoid the turnover, the resulting excitation energies will be closer to the exact result.^10^

Inspired by the promising results from xlinCCD(2) applied to the Hubbard model, we calculated BDEs for first–row transition metal diatomics of various correlation strengths, including metal hydrides, chlorides, and oxides. For these molecules, both xlinCCD(2) methods performed on par with linCCD and CCD. Interestingly, linLCCD­(hh) provided the overall smallest mean absolute error (Table S1). While K_2_, Zn_2_, and the closed-shell Ni_2_ and Cu_2_ can reasonably be treated with CCD and linCCD, qualitative accuracy for the remaining first–row transition metal homonuclear diatomics mandates *t*_1_ amplitudes as well as perturbative triples amplitudes, at minimum.^77^ With that in mind, it is encouraging that for the heteronuclear transition metal diatomics, xlinCCD(2)@linLCCD outperforms linCCD by 0.9 kcal/mol, coming within 0.3 kcal/mol of CCSD.

As a first foray into excited state properties with xlinCCD(2), we computed the first singlet–triplet (S-T) gaps via ΔMP and ΔCC methods for our new data set (TinySpins25) of 25 transition metal diatomics containing metals Ag, Au, Cd, Cu, Hg, Pt, Ru, Sc, and Zn. The theoretical best estimate (TBE) gaps for TinySpins25 are provided in the Supporting Information. The gaps are fairly small (averaging to 1.35 eV and ranging from 0.09 to 3.38 eV), making this a challenging data set for single-reference methods even though the *T*_1_-diagnostic^99^ never exceeds 0.05.^100^ The xlinCCD(2) methods are theoretically similar in computational cost to MP3 but provide much smaller errors for the gaps (Figure 4). For these equilibrium-geometry diatomics, linCCD provides excellent results within chemical accuracy. Even so, our bond dissociation curves (e.g., Figure 2) suggest that the xlinCCD(2) methods will out-compete linCCD by providing robust results not just at the equilibrium geometry but also during bond dissociation. The xlinCCD(2) correction reduces the S-T gap root-mean-square error to 0.25 eV, down from 0.40 eV for linLCCD (see Supporting Information). Similarly, the error in the xlinCCD(2)@linLCCD­(hh) gaps has been reduced to 0.27 eV, down from 0.41 eV for linLCCD­(hh) (see Supporting Information), so we can conclude that making the more affordable hole–hole approximation at the linLCCD step does not have much of an effect on the accuracy of the gaps.

**Figure 4:**
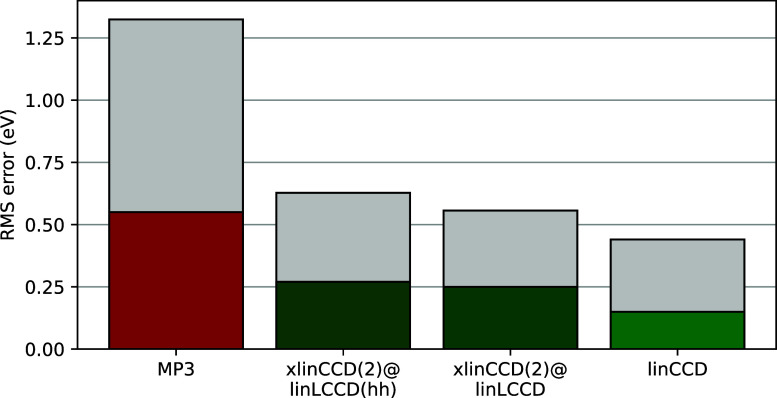
Root-mean-square error (eV) (colored bars) and maximum errors (gray bars) for the lowest energy singlet/triplet gaps of 25 transition metal diatomics as computed by ΔCC and ΔMP2 methods in the def2-QZVPPD basis.

Solvated Li^+^ clusters pose a surprisingly challenging problem for many quantum chemistry methods, providing an interesting, industrially relevant test system for our new xlinCCD(2) methods. Of the density functionals tested by Stevensen et al., the poor performance of B2PLYP-D3 (mean absolute error of 5.9 kcal/mol) relative to lower-rung functionals like ωB97X-D3­(BJ) and CAM-B3LYP-D3 on the problem of predicting association energies for Li^+^/ethylene carbonate (EC) clusters caught our attention. We suspect that the failure of B2PLYP-D3 is due to its dependence on the MP2 wave function, and our suspicion is corroborated by our finding that MP2 provides a mean absolute error of 10.5 kcal/mol for this problem (Table 2).

**Table 2:** Energies of Association of Li/EC Clusters in kcal/mol

benchmark			errors^a^						
#Li	#EC	CCSD(T)^b^	MP2	linLCCD(hh)	linLCCD	xlinCCD(2)^c^	xlinCCD(2)^d^	linCCD	CCD
1	1	–51.3	3.4	1.8	0.4	1.5	1.6	2.3	1.7
1	2	–90.1	5.6	2.8	0.4	2.2	2.4	2.9	0.9
1	3	–116.0	7.5	4.1	1.4	3.5	2.3	5.4	4.1
1	4	–129.9	2.0	1.6	9.7	8.8	8.5	6.4	7.5
2	4	–157.9	13.3	9.6	0.7	1.1	1.5	3.7	2.6
3	4	–101.1	17.6	13.0	2.9	3.9	4.3	7.0	5.2
4	6	–97.4	24.0	5.9					
		MAE	10.5	5.5	2.6	3.5	3.4	4.6	3.6

a All calculations done in the Def2-TZVPD basis set.

b DLPNO–CCSD­(T)/aug-cc-pVTZ data from.^81^

c linLCCD­(hh) reference wave function.

d linLCCD reference wave function.

Overall, we find that xlinCCD(2) provides results within our expectations for Li^+^/ethylene carbonate clusters. The hole–hole approximation to the linLCCD reference wave function has little effect on the accuracy of the xlinCCD(2) results, which are of CCD-level quality (within 0.1 kcal/mol of each other). Both reference wave functions for xlinCCD(2) also lead to 1 kcal/mol improvements over standard linCCD.

While likely not quite as affordable as MP2 even if implemented with our one-shot, memory-efficient strategy,^50^ linLCCD­(hh) (mean absolute error 5.5 kcal/mol) performs substantially better than MP2 (10.5 kcal/mol error), suggesting that a double hybrid functional based upon linLCCD­(hh) might be capable of providing better results for these lithium clusters. Such a density functional is forthcoming from our research group.^101^ In the immediate context, this result implies that linLCCD­(hh) wave functions are a better jumping-off point for higher-order perturbative corrections.

Finally, we have numerically verified the important property of size consistency for xlinCCD(2)@linLCCD and xlinCCD(2)@linLCCD­(hh) by considering the case of two hydrogen dimers. To see this analytically for the first method, suppose we simultaneously diagonalize *X*_*i*_^*j*^ and *X*_*b*_^*a*^ (*X*_*i*_^*j*^⊕*X*_*b*_^*a*^) so that eq 18 becomes

$$\delta t_{ij}^{ab}=-\frac{P_{ij}P_{ab}\left({\mathrm{t̃}}_{X_{im}^{ae}}{ṽ}_{ej}^{mb}\right)}{2{\mathrm{X̃}}_{b}-2{\mathrm{X̃}}_{j}}$$ 21

in the new basis ${\mathrm{X̃}}_{j}⊕{\mathrm{X̃}}_{b}$ of eigenvectors of *X*_*i*_^*j*^⊕*X*_*b*_^*a*^. Note that the block-diagonal basis transformation only mixes occupied orbitals with other occupied orbitals and likewise for virtual orbitals.

As linLCCD is size consistent, for any disjoint *i* → *a* and *m* → *e* excitation pairs localized on well-separated fragments, ${\mathrm{t̃}}_{X_{im}^{ae}}$ will be zero. Similarly, for any disjoint *j* → *b* and *m* → *e*, ${ṽ}_{ej}^{mb}$ will be zero. By transitivity, δ*t*_*ij*_^*ab*^ is zero for any disjoint *i* → *a* and *j* → *b* excitation pairs. A similar argument holds for xlinCCD(2)@linLCCD­(hh), since linLCCD­(hh) is also size consistent.^50^ (See Supporting Information.) We note that xlinCCD(2) is a coupled electron pair theory that contributes no correlation between excitation pairs on disjoint molecular fragments.

## Conclusion

In conclusion, we have presented a size-consistent, perturbative correction to linCCD called xlinCCD(2). Our approach can take any reference wave function as input, but we have tested the specific choices of linLCCD and linLCCD­(hh). Via calculations of thermochemical properties, bond dissociation energies and singlet–triplet gaps of transition metal diatomics, and the ozone asymmetric stretch vibrational mode, we have shown that xlinCCD(2) provides results of quality comparable to CCD. We also find that xlinCCD(2) produces CCD-quality results for strongly correlated systems well beyond the equilibrium geometry. xlinCCD(2) is capable of dissociating covalent bonds of homonuclear diatomics and performs well for the Hubbard model at high interaction strength. In general, xlinCCD(2) performs as well as linCCD at equilibrium but vastly outperforms it beyond the Condon region.

Next steps include algorithmic enhancements of the efficiency of xlinCCD(2) via one-shot implementations proposed in the Supporting Information. Apart from appearing more amenable to tensor hypercontraction density fitting algorithms than the usual iterative CC approaches,^102^ such one-shot algorithms could also avoid numerical instabilities that plague nonlinear amplitude equations.^103^ Given the clear importance of singles amplitudes in many of the chemical systems explored, we are currently formulating a related, *t*_1_-inclusive xCCPT approach. Overall, our results suggest it is possible to rescue single-reference linCCD approaches for strongly correlated systems.

## Supplementary Material











## Notes

Additional potential surfaces, thermochemistry data, and computational details for TinySpins25 data set (PDF)
Numerical PES data (XLSX)
W4-11 thermochemical data (XLSX)
BDEs, bond lengths, and spin states for first–row transition metal diatomics (XLSX)
S–T gaps and bond lengths for transition metal diatomics (TinySpins25) (XLSX)
