# Supplementary material for: The Dose Makes the Poison: Perturbative Steps toward the Ultimate Linearized Coupled Cluster Method
Source: J Chem Theory Comput. 2026 Apr 22;22(9):4452–61. doi: 10.1021/acs.jctc.6c00366 (PMC13178949; doi:10.1021/acs.jctc.6c00366)
Supplement: Supplementary file 1 [file ct6c00366_si_001.pdf]

Supporting Information for
The Dose Makes the Poison: Perturbative Steps Toward the Ultimate Linearized Coupled Cluster Method

Sylvia J. Bintrim, Ella R. Ransford, and Kevin Carter-Fenk*
Department of Chemistry, University of Pittsburgh, Pittsburgh, Pennsylvania 15218, USA
(Dated: April 6, 2026)

*kay.carter-fenk@pitt.edu

1. Mosaic-type terms in xlinCCD(2)

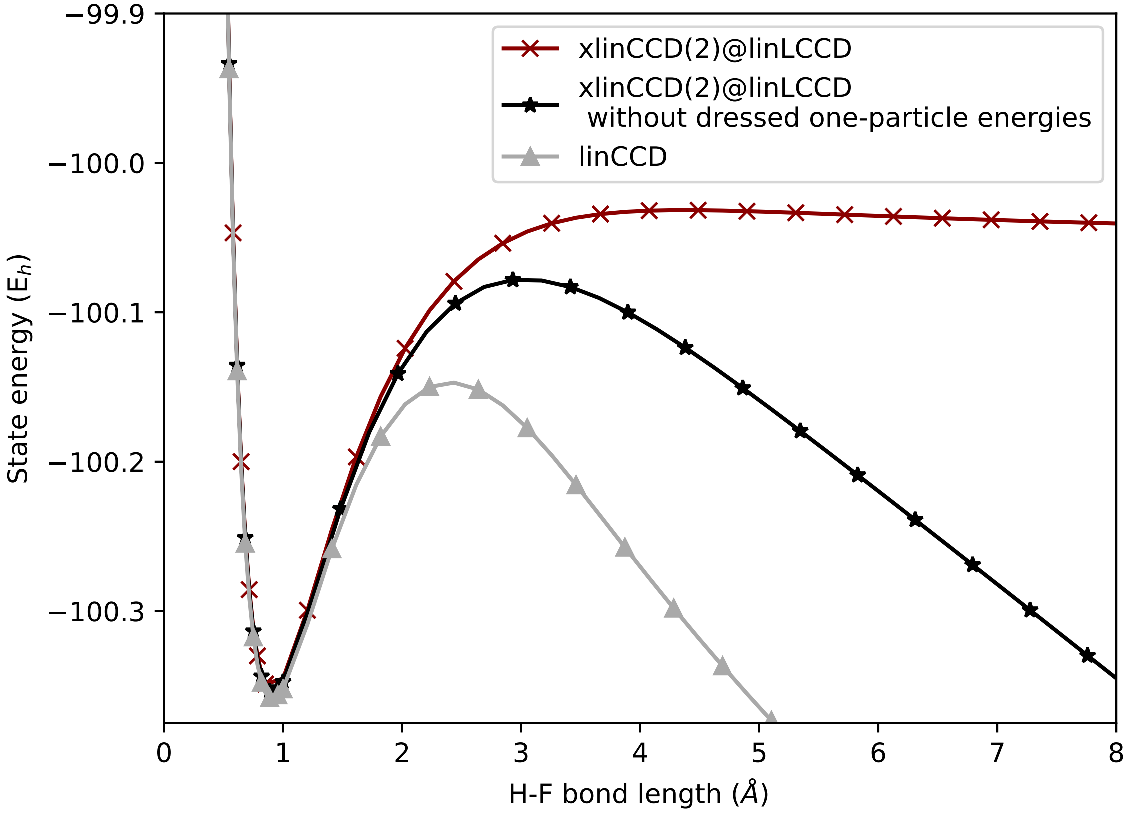

Figure S1. Ground state dissociation curves of FH in the aug-cc-pVTZ basis.

Figure S1 shows the importance of employing dressed one-particle energies in xlinCCD(2) for bond dissociation with restricted orbitals. Without this choice of $\hat{H}_0$ resulting in mosaic-type terms in the xlinCCD(2) equation, xlinCCD(2) still performs better than linCCD but also eventually diverges downward for bond dissociation.

2. Minimal Basis H_2

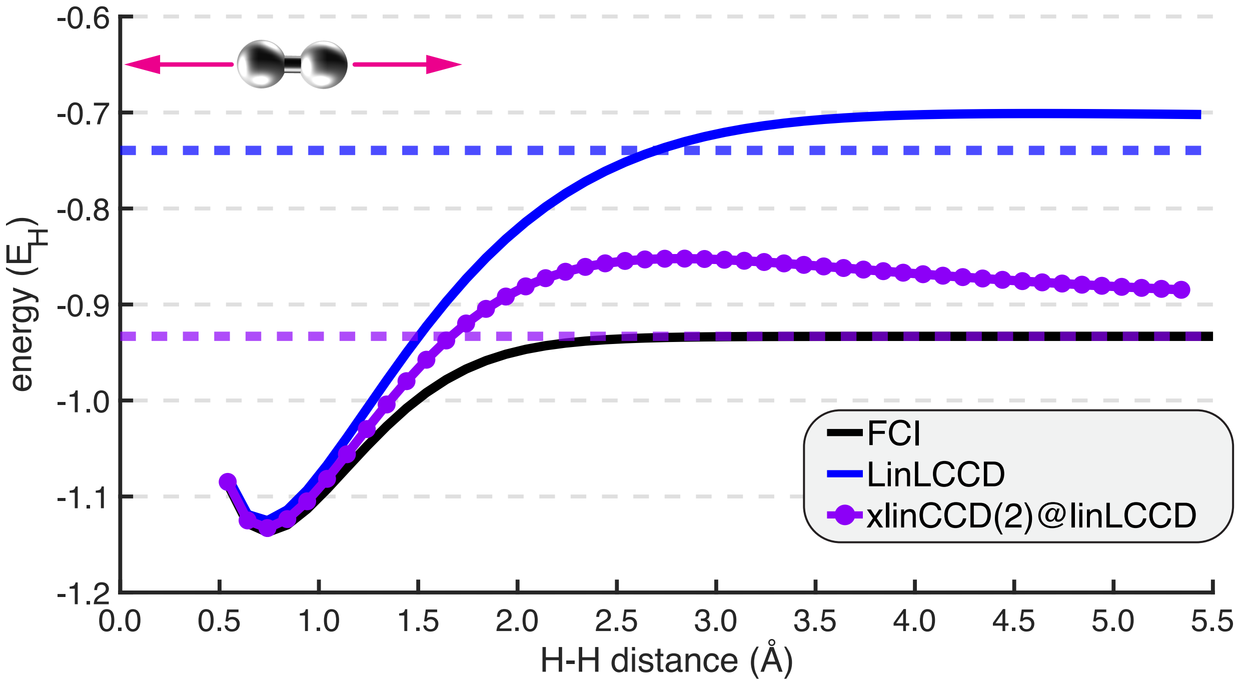

Figure S2. Restricted linLCCD and xlinCCD(2)@linLCCD potential energy surfaces for H_2 bond dissociation in the STO-3G basis compared with the (exact) CCSD solution. Dashed lines are estimates of the asymptotic limits calculated at 10,000 Å.

Figure S2 shows the bond dissociation potential energy surfaces obtained for minimal basis H_2. Critically, the xlinCCD(2)@linLCCD method produces the exact dissociation limit for this 2-electrons in 2-orbitals case. Other improvements in relative energy are seen across the entire potential surface when using xlinCCD(2)@linLCCD.

3. W4-11 Thermochemistry

For the 83 non-multi-reference bond dissociation processes in Figure S3, we see that none of the wave function methods assessed here provide BDEs within chemical accuracy (2 kcal/mol). Both varieties of xlinCCD(2) and linCCD gave somewhat more accurate BDEs than CCD. Together with our qualitatively correct dissociation curves, these results suggest that xlinCCD(2) provides results on par with CCD for bond-breaking processes.

MP2, linLCCD(hh), xlinCCD(2), and CCD provide overall W4-11 thermochemical data with substantial root-mean-square errors in the range of 6-8 kcal/mol. Interestingly, linCCD generally performs slightly better than the other methods. We conclude that xlinCCD(2) typically provides results of a quality comparable to those of linCCD or CCD for these weakly-correlated systems. Like other simplified or regularized methods tailored to the strong correlation problem,[3] the actual benefits of the xlinCCD(2) approach become more apparent for more challenging problems like bond dissociation curves.

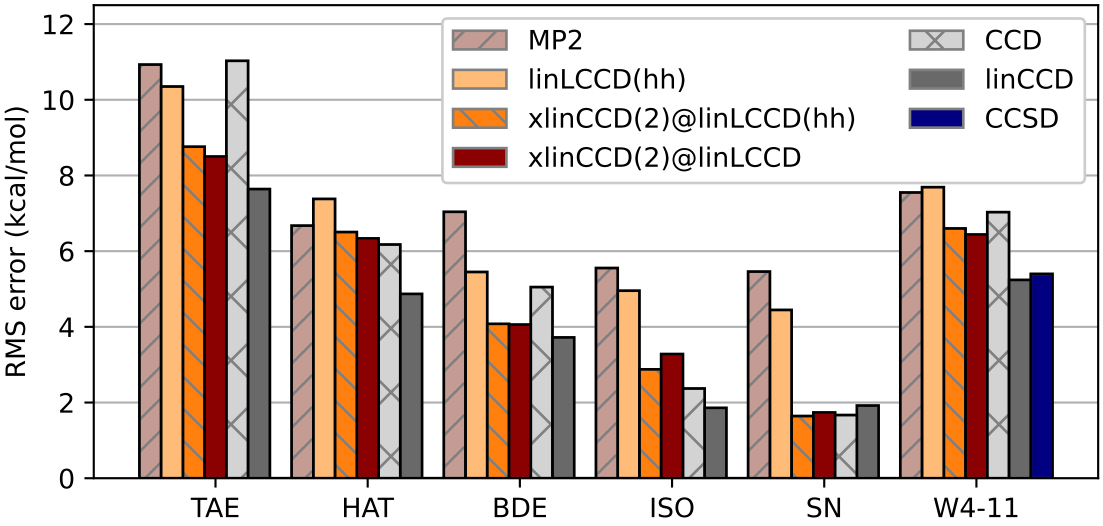

Figure S3. Root-mean-square errors (kcal/mol) produced by various wave function methods applied to subsets of thermochemical processes within W4-11: non-multireference total atomization energies (TAE), non-multireference heavy-atom transfer (HAT), non-multireference bond-dissociation energies (BDE), isomerization (ISO), and nucleophilic substitution (SN) energies. MP2 data is taken from Ref. 1 and CCSD data from Ref. 2.

4. BDEs of first-row transition metal heteronuclear diatomics

Table S1. Mean absolute errors (kcal/mol) in BDEs for first-row transition metal heteronuclear diatomics, computed using CC methods. Reference bond lengths, spin states, and BDEs are from Ref.s 4 and 5. Spin-orbit corrections from Ref. 5 were applied.

	linLCCD(hh)	linLCCD	xlinCCD(2)@ linLCCD(hh)	xlinCCD(2)@ linLCCD	linCCD	CCD	CCSD
hydrides	3.71	5.14	5.70	5.23	5.71	5.01	6.44
chlorides	7.81	6.10	5.20	5.20	4.17	4.47	5.31
oxides	11.01	25.05	19.61	16.96	19.28	22.03	14.54
all three	7.49	12.41	10.20	9.12	9.98	10.65	8.78

5. One-shot reformulation of xlinCCD(2)@linLCCD(hh)

Although we employ only the naïve implementation in this paper, change-of-basis implementations of linLCCD(hh) and xlinCCD(2)@linLCCD(hh) that are isomorphic with MP2 may reduce the methods' execution times. As detailed in Ref. 6, we can perform a memory-efficient linLCCD(hh) calculation via (1) an initial diagonalization of dressed Fock elements in the hole-hole space and (2) subsequent, one-shot, N^4-scaling perturbative step if we employ density fitting/resolution of the identity for the two-electron integrals.[6]

A similar re-formulation of xlinCCD(2) is possible on top of the linLCCD(hh) reference. First, we can simultaneously diagonalize the dressed one-particle energies X_i^j and X_b^a ($X_i^j \oplus X_b^a$):

$$X_b^a = f_b^a - \frac{1}{2} t_{\mathrm{X}mn}^{ae} v_{be}^{mn} \rightarrow \tilde{X}_b \tag{S1a}$$

$$X_i^j = f_i^j + \frac{1}{2} t_{\mathrm{X}im}^{ef} v_{ef}^{jm} \rightarrow \tilde{X}_j \tag{S1b}$$

Note that for the xlinCCD(2) correction on top of linLCCD(hh), we have

$$X_{ij}^{ab} = \frac{1}{2} t_{\mathrm{X}ij}^{ef} v_{ef}^{ab} + \mathcal{P}_{ij}\mathcal{P}_{ab}(t_{\mathrm{X}im}^{ae} v_{ej}^{mb}) \tag{S2}$$

as we must include the linear, particle-particle ladder term as well as the linear ring/crossed ring terms in the perturbative step.

Next, we can perform the one-shot, N^6-scaling xlinCCD(2) correction in the basis of eigenvectors of $X_i^j \oplus X_b^a$, denoted by tilde symbols:

$$\delta t_{ij}^{ab} = \frac{\frac{1}{2}\tilde{t}_{\mathrm{X}ij}^{\ ef}\tilde{v}_{ef}^{ab} + \mathcal{P}_{ij}\mathcal{P}_{ab}(\tilde{t}_{Xim}^{\ ae}\tilde{v}_{ej}^{mb})}{2\tilde{X}_b - 2\tilde{X}_j} \tag{S3}$$

While CCD, linCCD, and xlinCCD(2)@linLCCD require iterative solution, this one-shot reformulation of xlinCCD(2) @linLCCD(hh) may have lower computational execution time.

6. TinySpins25 Data Set

The TinySpins25 data set contains estimates of the lowest-energy singlet-triplet gap for 25 heteronuclear transition-metal diatomics. We optimized the bond lengths at the ωB97M-V/Def2-TZVPP level of theory. The CCSDT(Q)$_\Lambda$ calculations were extrapolated to the complete basis set limit with a two-point extrapolation scheme using the Def2-SVPD and Def2-TZVPPD basis sets. We applied the same frozen core scheme as Ref. 7 for 3d metals and applied the pseudopotential that was optimized for the Karlsruhe basis sets for all 4d and 5d metal atoms. When pseudopotentials were applied, only core orbitals on the non-metal atom were frozen. We tested this CBS extrapolation scheme by comparing our results for RuC with the high-level (aug-cc-pV5Z-PP) multireference configuration interaction (MRCI) data from Ref. 8. Their MRCI/aug-cc-pV5Z-PP data are in excellent agreement with experiment and their estimated gap is 2.1 kcal/mol. Our CCSDT(Q)$_\Lambda$/CBS result is 2.0 kcal/mol, which is in near-perfect agreement with large-basis MRCI. Our triple-ζ estimates for the gaps of ScH, ScF, CuH, CuF, CuCl, ZnO, and ZnS (the subset of TinySpins25 that overlaps with Quest #8)[9] are also within 1 kcal/mol of the aug-cc-pVTZ Quest #8 theoretical best estimates (which often equates to FCI). Overall, we estimate that the accuracy of our predicted gaps is less than 1 kcal/mol from the true non-relativistic values. It is our intention to supply the community with a non-relativistic estimate of all of these gaps (save the relativistic effects that are inherent to the Karlsruhe pseudopotentials), so TinySpins25 does not make use of scalar nor vector relativistic corrections.

Finally, we note that no T_1 diagnostic on any system in TinySpins25 exceeds 0.05, implying that this may be a good test set to benchmark density functionals due to its single-reference character. However, further assessments of the multiconfigurational nature of these small-gap systems would be necessary to make that determination.

---

[1] Carter-Fenk, K.; Shee, J.; Head-Gordon, M. Optimizing the regularization in size-consistent second-order Brillouin-Wigner perturbation theory. *J. Chem. Phys.* **2023,** *159,* 171104:1–8.

[2] Lee, J.; Pham, H. Q.; Reichman, D. R. Twenty years of auxiliary-field quantum monte carlo in quantum chemistry: An overview and assessment on main group chemistry and bond-breaking. *Journal of Chemical Theory and Computation* **2022,** *18,* 7024–7042.

[3] Wang, Z.; Shen, Y.; Head-Gordon, M. Third-order perturbation theory made regular: A noniterative correction to the size-consistent second-order brillouin–wigner perturbation theory. *J. Phys. Chem. Lett.* **2026,** .

[4] Furche, F.; Perdew, J. P. The performance of semilocal and hybrid density functionals in 3d transition-metal chemistry. *J. Chem. Phys.* **2006,** *124,* 044103:1–27.

[5] Zamani, A. Y.; Zulueta, B.; Ricciuti, A. M.; Keith, J. A.; Carter-Fenk, K. Kohn-Sham density encoding rescues coupled cluster theory for strongly correlated molecules. *arXiv preprint https://arxiv.org/abs/2602.06149* **2026,** .

[6] Carter-Fenk, K. Diagrammatic simplification of linearized coupled cluster theory. *J. Phys. Chem. A* **2025,** *129,* 7251–7260.

[7] Hait, D.; Tubman, N. M.; Levine, D. S.; Whaley, K. B.; Head-Gordon, M. What levels of coupled cluster theory are appropriate for transition metal systems? A study using near-exact quantum chemical values for 3d transition metal binary compounds. *J. Chem. Theory Comput.* **2019,** *15,* 5370–5385.

[8] Tzeli, D.; Karapetsas, I. Quadruple bonding in the ground and low-lying excited states of the diatomic molecules TcN, RuC, RhB, and PdBe. *J. Phys. Chem. A* **2020,** *124,* 6667–6681.

[9] Jacquemin, D.; Kossoski, F.; Gam, F.; Boggio-Pasqua, M.; Loos, P.-F. Reference vertical excitation energies for transition metal compounds. *J. Chem. Theory Comput.* **2023,** *19,* 8782–8800.
